# The Great Imposter: An Atypical Case of Pleomorphic Adenoma With Review of Literature

**DOI:** 10.7759/cureus.52851

**Published:** 2024-01-24

**Authors:** Pranjal Rai, Arpita Sahu, Neha Mittal, Vasundhara Patil

**Affiliations:** 1 Department of Radiology, Tata Memorial Hospital, Mumbai, IND; 2 Department of Pathology, Tata Memorial Hospital, Mumbai, IND

**Keywords:** fine needle aspiration, head and neck tumors, slow-flow vascular malformation, multicentric pleomorphic adenoma, pleomorphic adenoma

## Abstract

Pleomorphic adenomas (PA) are the most common type of salivary gland tumors. These slow-growing benign tumors most commonly involve the parotid gland, but can sometimes occur at atypical sites such as the submandibular or minor salivary glands. We describe an atypical case of pleomorphic adenoma with multicentric involvement of the parotid, the submandibular gland, and the parapharyngeal space in a 35-year-old male which mimicked a slow-flow malformation on magnetic resonance imaging (MRI). Diagnosis was confirmed on fine needle aspiration cytology, and conservative approach was opted for the patient in view of perioperative risks. This case highlights the uncommon multicentricity and atypical presentation of PA, challenging the initial differential diagnosis based on MRI features. It also underscores the importance of considering atypical presentations and utilizing accurate diagnostic tools like cytology for managing complex salivary gland tumors.

## Introduction

Pleomorphic adenomas (PA) represent the predominant type of salivary gland tumors, comprising up to two-thirds of all cases [1]. These tumors are also referred to as benign mixed tumors (BMT) because of their unique dual origin, encompassing both epithelial and myoepithelial components. Characterized by their painless nature and slow growth, PA are well-defined and usually encapsulated in major salivary glands, distinguishing them from their minor gland counterparts. Development of PA has been linked to prior head and neck irradiation in published literature [2]; however, most of these are sporadically occurring.

While the parotid gland is the most commonly affected site, PA can occasionally manifest in the submandibular and minor salivary glands [1]. These have a low risk of malignant transformation, which is in proportion to the time the lesion is in situ [3]. The most reliable method of PA diagnosis is typically core needle biopsy [4]. The treatment of choice for the management of PA is usually wide excision with negative margins [5]. Local site recurrence is rare but has been described in cases with intraoperative tumor spillage or inadequate surgical resection. For inoperable cases, radiation therapy is employed [6].

We describe an atypical case where a PA exhibited a remarkable presentation, spanning the entire right side of the neck, with the major bulk of disease in the submandibular and parotid glands with discrete lobulated areas in the retropharyngeal region, extending along the sternocleidomastoid muscle to the base of the neck and another focus seen in the midline in the submental location. This unique presentation bore a striking resemblance to a slow-flow vascular malformation or a large plexiform neurofibroma.

This report highlights the diagnostic challenges posed by such an unusual multicentric presentation of PA and the value of techniques like fine needle aspiration cytology (FNAC) in such cases. Moreover, informed consent was obtained from the patient to publish this case report and any accompanying images, in accordance with institutional and ethical guidelines.

## Case presentation

A 35-year-old male presented with swelling posterior to the angle of the mandible and right neck for the past four to five years. The patient had a past history of tuberculosis 20 years ago and had received treatment for the same. He had no clinical comorbidities. On examination, there was a diffuse firm, non-tender swelling posterior to the right angle of the mandible of approximately 5x5 cm size. Palpable right level IB, II, and III nodes were also noted. An ultrasound of the neck was done which revealed multiple, lobulated, hypoechoic lesions in the right neck, along IA, IB, and II nodal groups. 

**Figure 1:**
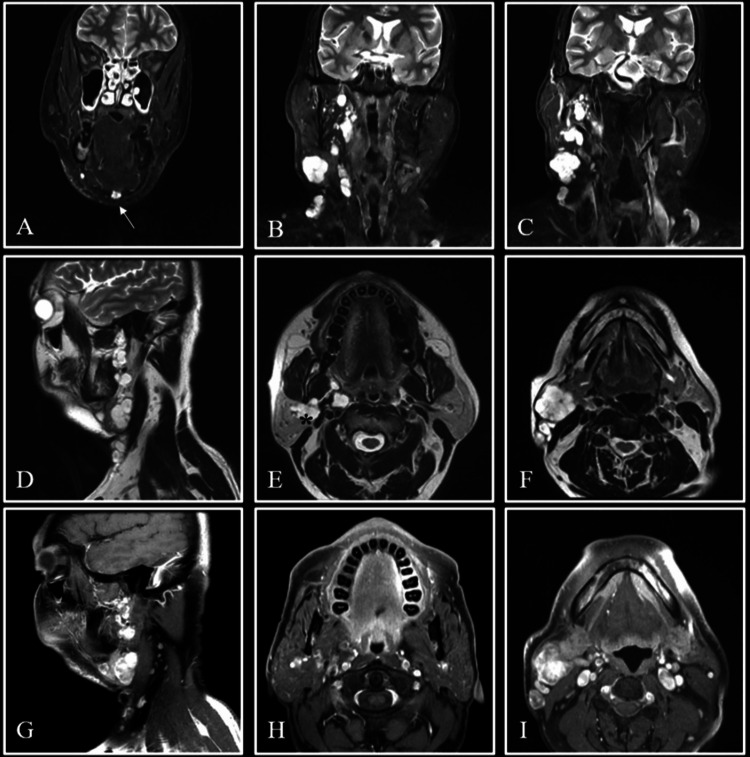
Imaging findings. Coronal STIR images (A, B, and C) show hyperintense lobulated lesions spanning the entire right side of the neck, extending from the base of the skull to the submandibular region inferiorly, and in the submental region (arrow). Sagittal (D) and axial (E and F) T2W images show multiple hyperintense lesions of similar morphology, involving the right side of the neck, the deep lobe of the parotid (asterisk), and the right submandibular gland. Post-contrast T1W images (G, H, and I) demonstrate enhancement within these lesions. STIR: short tau inversion recovery; T2W: T2-weighted; T1W: T1-weighted

A magnetic resonance imaging (MRI) scan was then ordered for the patient to better delineate the extent of the disease, which revealed multiple, discrete, and irregular well-defined lobulated soft tissue lesions spanning the entire length of the neck on the right side, more specifically the superficial and deep lobes of the parotid, the right parapharyngeal space, the high infratemporal fossa, the right submandibular gland, and the submental region, with few extending to the midline. The lesions were isointense on T1-weighted images and hyperintense on T2-weighted images and showed heterogeneous post contrast enhancement. No evidence of blooming on susceptibility-weighted imaging (SWI) or diffusion restriction was noted. A few of the lesions also showed smooth peripheral enhancement (Figure 1).

**Figure 2:**
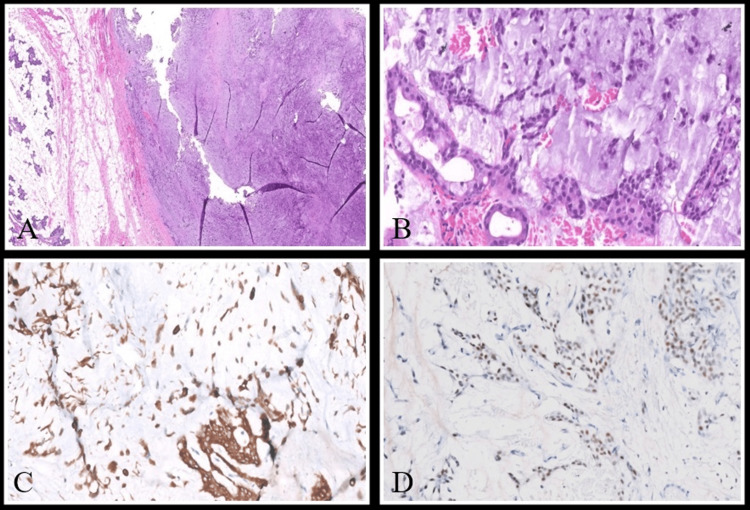
Histopathological and immunohistochemical findings. A: A well-circumscribed tumor involving the salivary gland (×40, H and E). B: On higher power, the tumor is composed of a monomorphic biphasic population of polyhedral epithelial and spindle-shaped/angular myoepithelial cells, in a background of hyalinized areas representing chondromyxoid stroma (×200, H&E). C and D: Immunohistochemistry shows the epithelial cells to be positive for CK7 (C, ×100, DAB), and the myoepithelial cells are positive for SOX10 (D, ×100, DAB). H&E: hematoxylin and eosin; CK7: cytokeratin-7; SOX10: SRY-box transcription factor 10; DAB: 3,3'-diaminobenzidine

The most likely differential for this case was given as a slow-flow venous malformation in the parapharyngeal region or a plexiform neurofibroma. FNAC from the aforementioned lesion was performed, which was positive for a pleomorphic adenoma. Additional biopsy from the right submandibular gland was also done, which showed a low-grade salivary neoplasm arranged in clusters and tubules (Figure 2).

The case was discussed in the multidisciplinary tumor board, and a conservative approach was opted for due to the multifocality, multicentricity, and operative challenge associated with the disease burden. Close follow-up was offered to the patient in view of the risk of malignant transformation. The patient remained on observation and the disease burden remained stable 18 months post diagnosis.

## Discussion

There are several distinct histologies and sites for salivary gland neoplasms. These involve not only the major salivary glands such as the parotid, submandibular gland, and sublingual gland but minor salivary glands as well. Approximately 9-14% of all salivary gland tumors occur within the minor salivary glands [7]. There is a near similar incidence of both benign and malignant tumors within the minor salivary glands with benign tumors being slightly more frequent [8].

PA are the most prevalent type of salivary gland tumor and can affect both the major and minor salivary glands. Around 10% of the PA tend to occur in the minor salivary glands, out of which the palate is the most common site, followed by the lip [9,10]. These subtypes of salivary gland tumors are also known as benign mixed subtypes and occur in females predominantly in their third to fifth decades [11]. The presentation in our case was atypical in a 35-year-old male.

PA on MRI are T1 isointense to hypointense and T2 hyperintense and show progressive and homogeneous post contrast enhancement. They don't show any restricted diffusion on diffusion-weighted imaging (DWI). Bony erosion and remodeling may rarely occur due to mass effect, which may be better highlighted on computed tomography (CT).

The most common differentials for tumors in this location are a slow-flow vascular malformation or a plexiform neurofibroma.

Out of all the vascular malformations, the venous malformations tend to be the commonest, ranging from small, circumscribed lesions to large infiltrative masses. These are essentially thin-walled, dilated vascular channels with deficient smooth muscle and normal endothelium lining that can vary in size and thickness [12]. They frequently cross the deep fascial planes of the head and neck [13]. MRI demonstrates these as venous lakes with an isointense signal on T1- and a homogeneously high signal on T2-weighted sequences, which show prominent enhancement on post-gadolinium T1-weighted images. They may often be seen tracking along muscle groups or nerves [13]. On T1- and T2-weighted images, phleboliths may appear as low signal foci within these venous lakes.

Plexiform neurofibromas are uncommon peripheral nerve sheath tumors occurring exclusively in patients with neurofibromatosis type 1. These arise due to the disorderly proliferation of all the neural elements of a peripheral nerve, which are interwoven (hence the term "plexiform"). These can be infiltrating masses especially when arising in closed spaces of the head and neck [14]. MRI demonstrates heterogeneously hyperintense infiltrating masses on T2/short tau inversion recovery (STIR) sequences, with tubular internal foci. Lesions may reveal the classical "target sign" of central hypointensity with a surrounding hyperintense signal on T2-weighted sequences, which corresponds to a dense central area of stromal collagen and a peripheral area of myxomatous tissue [14,15]. Mild post contrast enhancement may be seen.

The differential diagnoses in our case were narrowed down to a slow-flow venous malformation, followed by plexiform neurofibroma and less likely PA, owing to its indistinguishable imaging characteristics as all of these are hyperintense on T2 and STIR sequences. The diagnostic dilemma was solved with the use of FNAC, which showed moderately cellular smears showing singly scattered and small clusters of epithelial and myoepithelial cells in a chondromyxoid stroma, thus clinching the final diagnosis of PA.

Unique features of our case include multicentricity, atypical presentation in the parapharyngeal space, and the clinical conundrum in characterizing the lesion. Cases of concurrent PA occur rarely; only 23 cases have been reported so far in the literature. Of these, only one case by Ladeinde et al. has been documented to involve both a major and minor salivary gland (in the parapharyngeal space) [16]. There is the involvement of both the right parotid and right submandibular gland, in addition to the involvement of the parapharyngeal space in our case, which is a very rare occurrence.

De novo involvement of the parapharyngeal space in PA is also a rare occurrence and usually presents with larger masses. Few cases reported until now have demonstrated the origin of PA from epithelial cell rests of salivary tissue in the parapharyngeal space [17,18]. Unlike other sites, the PA along the parapharyngeal space may also present with atypical symptoms like trismus, neuralgia, palsies of the ninth, 10th, or 11th cranial nerves, or otalgia. The most common presentation is that of benign parapharyngeal submucosal swelling along the lateral pharyngeal wall. Our patient did not have any of these symptoms, likely due to the smaller size of these masses. This also tipped the differential diagnosis in our case more in favor of a low-flow venous malformation.

FNAC is a preferable and reliable problem-solving tool in such cases and helped in providing us with an accurate diagnosis. The biopsy yielded crucial additional insights, including the tumor's immunohistochemical profile.

Management of these parapharyngeal tumors is primarily surgical; however, there is a risk of incomplete resection due to the complex anatomical location, and hence, close follow-up is of paramount importance [19]. Another important post-surgical complication is injury to nerves and vessels and bleeding from the base of the skull. Considering these risks, the multicentricity of the disease, and significant perioperative morbidity, a conservative approach was adopted in our case, in the form of a six-monthly MRI to look for disease progression.

## Conclusions

PA is a type of benign mixed tumor which can have rare involvement of parapharyngeal salivary glands and can have a broad radiological differential diagnosis based on its imaging features. We have presented an atypical case of multifocal and multicentric PA mimicking a slow-flow venous malformation on MRI. We have also highlighted the use of FNAC as a problem-solving tool to accurately provide us with the diagnosis and to avoid interventions such as excision biopsies which can cause significant perioperative morbidity as well as neurovascular complications.
